# Verheerender Verlauf – gutes Ende! Exzellente Ergebnisse dank Implantation eines künstlichen Blasenschließmuskels trotz perinealer Urethrostomie – ein Fallbericht

**DOI:** 10.1007/s00120-025-02711-5

**Published:** 2025-10-10

**Authors:** Niklas Matthias Bohne, Roberto Olianas, Arne Strauß, Mirjam Naomi Mohr, Lutz Trojan, Mathias Reichert

**Affiliations:** Klinik für Urologie, Universitätsmedizin Göttingen, Göttingen, Deutschland

**Keywords:** Postprostatektomieinkontinenz, Harnstressinkontinenz, AMS-800, Inkontinenztherapie, Boutonnière, Postprostatectomy incontinence, Urinary stress incontinence, AMS-800, Incontinence therapy, Boutonnière

## Abstract

Dieser Fall beschreibt zum ersten Mal die Implantation eines AMS-800©-Sphinkters (Boston Scientific Corporation, Marlborough, MA, USA) bei einem Patienten mit Belastungsinkontinenz nach radikaler Prostatektomie, der durch komplizierten Verlauf der vorherigen Inkontinenztherapie letztlich mit einer perinealen Urethrostomie versorgt wurde. Die Kontinenzsituation und die postoperative Lebensqualität nach Versorgung mit dem neu implantierten Sphinktersystem zeigen, dass eine Boutonnière keine Kontraindikation für die Versorgung mittels künstlichem Blasenschließmuskel darstellt.

## Abstract (en)

In this case report, we describe, for the first time, the implantation of an AMS-800 sphincter (Boston Scientific Corporation, Marlborough, MA, USA) in a patient with stress incontinence following radical prostatectomy, who was ultimately treated with an perineal urethrostomy due to a complicated course of previous incontinence therapy. The continence status and postoperative quality of life after treatment with the newly implanted sphincter system clearly demonstrate that a boutonnière deformity is not a contraindication for treatment with an artificial urinary sphincter.

## Anamnese

Der Fall handelt von einem 72-jährigen Patienten nach radikaler Prostatektomie (T2c, N0, M0, R1, GS7a, aktueller PSA 0,01 ng/ml), welcher im Vorfeld bei schwerer postoperativer Belastungsinkontinenz mittels artifiziellem Sphinkter mit distalem Doppel-Cuff (AMS-800 ©, Boston Scientific, Marlborough, MA, USA) versorgt wurde (2 × 4,5-cm-Manschetten). Ambulant kam es jedoch wohl zu einem subakuten Harnverhalt. Daher wurde im ambulanten Bereich ein 18-CH-Dauerkatheter (DK) eingelegt und für 2 Wochen belassen.

**Abb. 1:**
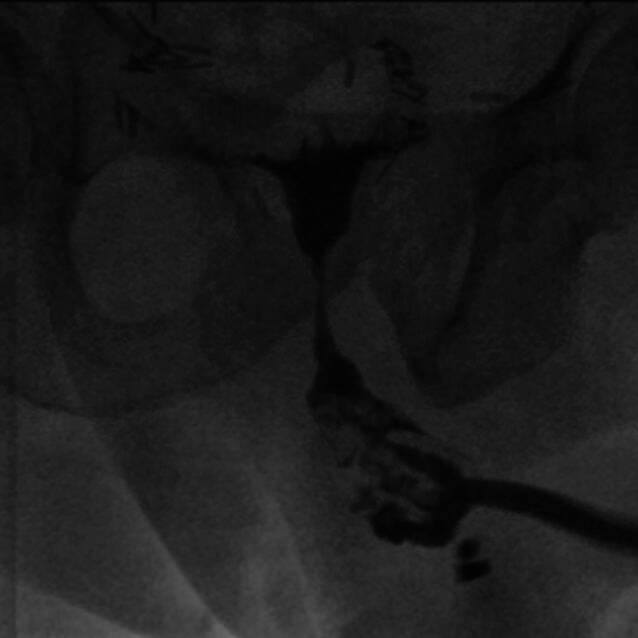
Retrograde Urethrographie mit Nachweis einer Harnröhrenarrosion

Anschließend stellte sich der Patient in einer ausgeprägten Infektsituation mit Fieber und Schmerzen in unserer urologischen Poliklinik vor. Der DK wurde entfernt, eine retrograde Urethrographie (Abb. 1) durchgeführt und nach Diagnosestellung einer Harnröhrenarrosion das Sphinktersystem explantiert. Intraoperativ zeigte sich eine komplette Nekrose der distalen, bulbären Harnröhre. Die notfallmäßige Versorgung beinhaltete neben der Explantation des gesamten Sphinktersystems die Anlage einer perinealen Urethrostomie und Einlage eines transurethralen DK.

Nach primärer Heilung ohne weitere Intervention war der Patient 8 Monate nach Explantation durchlaufinkontinent und wünschte eine Therapie der Inkontinenz.

## Klinischer Verlauf

Aufgrund der weiterhin bestehenden schweren Inkontinenz nach Explantation des artefiziellen Sphinkters und Anlage der Boutonnière besteht der ausdrückliche Wunsch des Patienten zur operativen Therapie der Belastungsinkontinenz. Aufgrund der anatomischen Lage des Neomeatus entwickelte sich trotz guter Wundheilung eine ausgeprägte Dermatitis durch den Urin, der sich neben dem DK entleerte.

Diese Situation zwang zu einer operativen Lösung des Problems. Zur Debatte standen eine supravesikale Harnableitung (z. B. Mitrofanoff-Vesikostomie mit simultanem Blasenhalsverschluss) und eine Reimplantation eines künstlichen Blasenschließmuskels. Nach Aufklärung, dass bislang kein Fall beschrieben wurde, der über eine Implantation eines AUS („artificial urinary sphincter“) bei Vorhandensein einer Boutonnière berichtet, entschlossen wir uns zu dem Versuch einer erneuten AUS-Implantation.

Durch Kapazitätsmessung ist weiterhin eine ausreichende Blasenkapazität nachgewiesen (ca. 550 ml). Sechs Monate nach Explantation erfolgt der Eingriff komplikationslos. Aufgrund der Vorgeschichte werden Reservoir (intraperitoneal) und Pumpe (skrotal) linksseitig implantiert. Die proximale Harnröhre zeigt sich vital und suffizient weit. Um die Perfusion der Harnröhre, welche nun unidirektional (antegrad) perfundiert wird, nicht zu kompromittieren, wird die Präparation mit äußerster Sorgfalt unternommen. Des Weiteren wird die Manschette locker adaptiert um die Harnröhre gelegt (6 cm) und weit nach proximal/membranös lokalisiert, wie erstmals von Schreiter beschrieben [9], um beim Sitzen keine störende Empfindung zu verursachen oder das System durch Druck zu gefährden.

Nach unkompliziertem postoperativem Verlauf mit zeitgerechter Wundheilung, DK-Entfernung nach 3 Tagen und Sphinkteraktivierung zeigt sich nach 12 Monaten eine Restinkontinenz am Tage mit dem Verbrauch von 3 Vorlagen unter Belastung. Nachts ist der Patient komplett trocken. Der Patient ist mit dem Zustand bereits zufrieden, jedoch im täglichen Leben durch die Restinkontinenz weiterhin eingeschränkt. Er beschreibt, dass das Sphinktersystem nicht störe und er es nicht spüre – auch nicht in sitzender Position.

Aufgrund des guten postoperativen Verlaufs und der aktuellen Kontinenzsituation, die verbesserungswürdig erscheint, wurde die Indikation zum Cuff-Downsizing gestellt.

## Operation

**Abb. 2:**
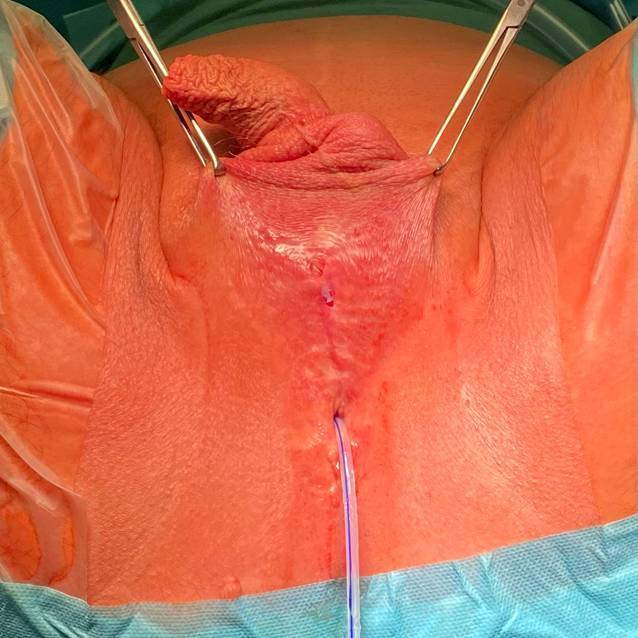
Präoperativer Status

Die Abb. 2 zeigt den präoperativen Status mit reizlosen Wund- und Hautverhältnissen. Der Sphinkter wird deaktiviert, nachdem die Manschette durch 3 Pumpenschläge entleert wurde.

**Abb. 3:**
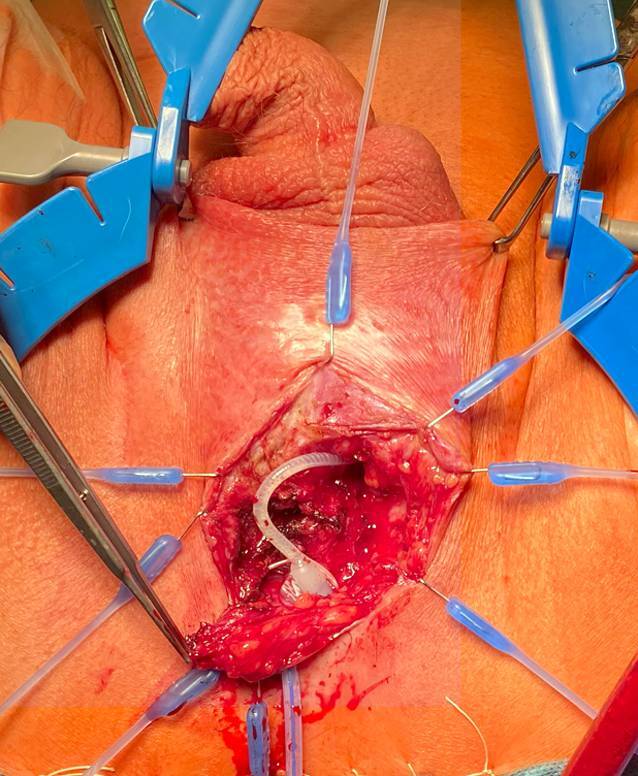
Manschettenschlauch und ventraler Anteil der Manschette. Harnröhre nach dorsokaudal luxiert

Nach medianer Inzision perineal wird am Übergang der Symphyse der Schlauch zur Manschette aufgesucht und identifiziert. Entlang des Schlauchverlaufs kann zur Manschette präpariert werden. Es wird penibel darauf geachtet, so wenig wie möglich zu präparieren, um die Harnröhrenversorgung nicht zu verschlechtern. Hierzu muss kutan der Schnitt nach links lateral der Harnröhre erweitert werden. Abb. 3.

**Abb. 4:**
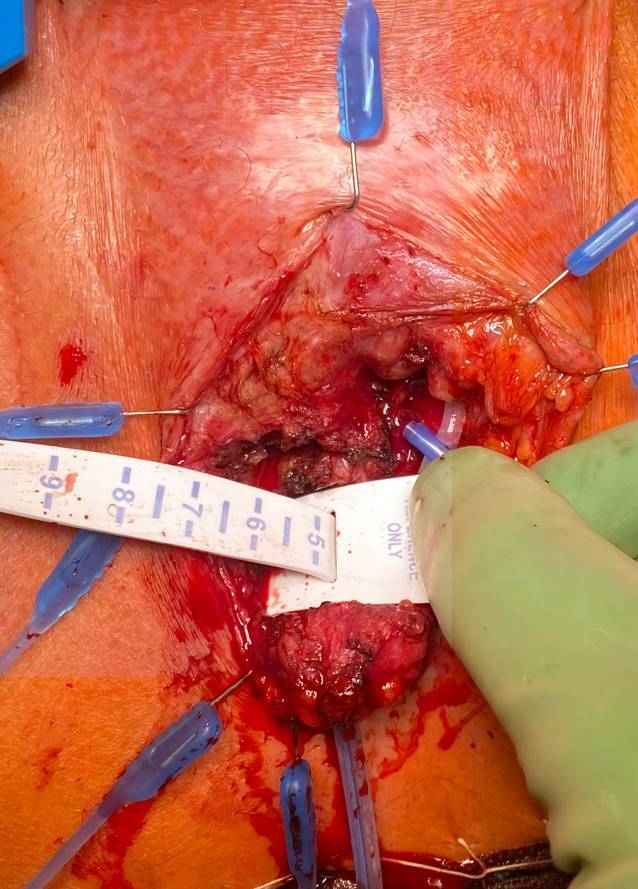
Ausmessen der Größe der neuen Manschette

Die Verschlussöse der Manschette wird durchtrennt und mittels des Maßbands die neue Manschettengröße (5 cm) bestimmt. Auch hier wird darauf geachtet, dass die Manschette locker adaptiert erscheint (Abb. 4).

**Abb. 5:**
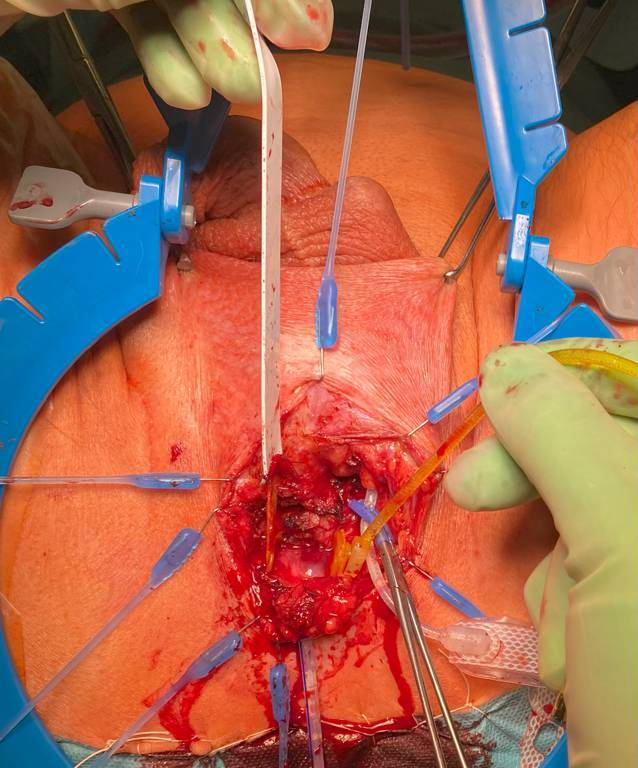
Durchzug der neuen Manschette (5 cm) und Platzierung in alter Position

Anschließend wird, ohne die Harnröhre zirkulär mobilisiert zu haben, die neue Manschette mittels Fixierung an das Maßband in die alte Position gebracht (Abb. 5).

Nachdem die neue Manschette positioniert ist, erfolgt der Zugang im linken Unterbauch. Die alte Manschette wird gekappt und eine Verbindung der neuen Manschette mittels Quick Connector an das alte System hergestellt.

**Abb. 6:**
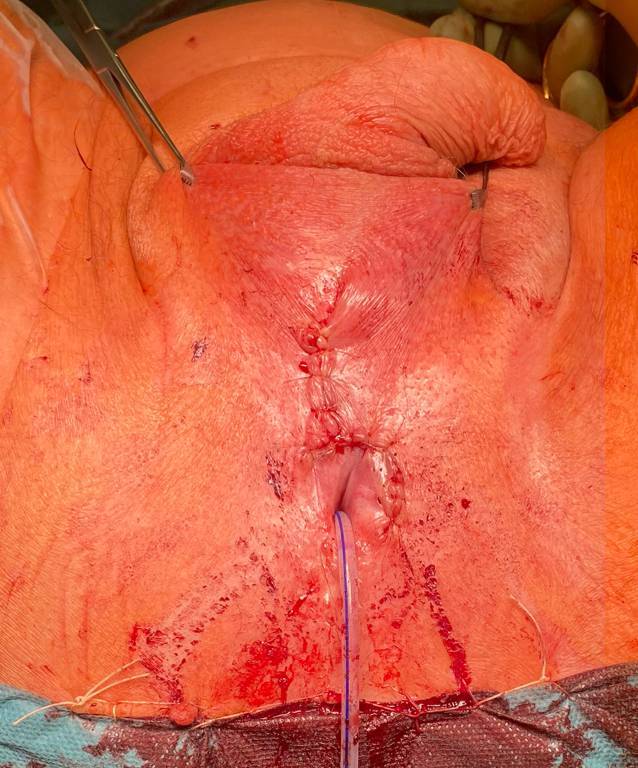
Postoperativer Situs

Die korrekte Funktion des Systems wird überprüft und bestätigt. Anschließend wird die Pumpe bei leerer Manschette deaktiviert und die Wunden verschlossen (Abb. 6).

## Postoperativer Verlauf und Ergebnis

Der transurethral einliegende Katheter konnte am 1. postoperativen Tag entfernt werden. Nach Katheterentfernung entleert der Patient die Harnblase bei bekannter Inkontinenz restharnfrei. Nach Aktivierung am 2. postoperativen Tag ist der Patient subjektiv zufrieden und weist eine komplette Kontinenz auf. Zur Objektivierung der Kontinenzsituation wird ein PAD-Test durchgeführt mit Verlust von maximal 2 g Urin nach körperlicher Anstrengung. Während des unmittelbaren postoperativen Verlaufs benutzt der Patient eine Sicherheitsvorlage, welche jedoch trocken bleibt. Der Patient wird nach Standardantibiose am 4. postoperativen Tag ohne Vorlagennotwendigkeit entlassen. Drei Monate nach Operation ist die Kontinenzsituation gleichbleibend.

## Diskussion

Dieser Fallbericht beschreibt erstmalig den Einsatz eines künstlichen Blasenschließmuskels bei Vorliegen einer Urethroperineostomie. Durch den erfolgreichen und befriedigenden Verlauf kann die Indikationsstellung der AUS-Implantation revolutioniert werden.

Die Postprostatektomieinkontinenz (PPI) ist eine relevante Nebenwirkung der kurativen Therapie des Prostatakarzinoms mittels radikaler Prostatektomie. Trotz immer besser werdendem Verständnis des Kontinenzmechanismus und Operationsmethoden ist es nicht gelungen, diese lebenseinschränkende Situation zu vermeiden. So liegt das Risiko einer PPI weiterhin zwischen 10 und 20 % [1].

Im Laufe der Zeit etablierten sich verschiedene Implantate in der operativen Therapie der PPI. Der künstliche Blasenschließmuskel, welcher 1972 eingeführt wurde, stellt heutzutage die Primärwahl der Therapie bei moderater und schwerer PPI dar [2]. Neben dem ZSI-375©-Sphinkter ist das bis zum heutigen Tage etablierte und unveränderte AMS-800©-Model das bislang einzige Model mit akzeptablen Langzeitergebnissen [3].

So wurde auch in dem hier beschriebenen Fall die Indikation zu einer AUS-Implantation bei schwergradiger Belastungsinkontinenz gestellt.

Ein Harnverhalt bei einliegendem Sphinktersystem ist eine bekannte Komplikation, welche in bis zu 31 % aller Fälle auftreten kann [4]. Unmittelbar nach DK-Entfernung – bei noch nicht aktiviertem System – ist dies meist durch eine postoperative Schwellung im Operationsgebiet bedingt. Die konservative Therapie besteht, neben einer antiphlogistischen Medikation, in der vorsichtigen Einlage eines transurethralen 12-Ch-Katheters. Leider kam es in unserem Fall durch den Einsatz eines 18-Ch-Katheters und langer Liegedauer zu einer Harnröhrenarrosion mit nachfolgender Nekrose der Harnröhre.

In der beschriebenen Notfallsituation blieb keine Alternative zur Explantation des gesamten Systems. Durch die Nekroseabtragung musste die distal-bulbäre Harnröhre komplett exzidiert und eine Boutonnière angelegt werden.

Durch die weiterhin bestehende schwergradige Belastungsinkontinenz wurde die Indikation zur erneuten Implantation eines AMS-800©-Sphinkters gestellt mit Implantation der Manschette proximal/membranös, als Alternative zur Harnableitung.

Eine mögliche Komplikation ist die insuffiziente Durchblutung der Harnröhre, da diese bei perinealen Urethrostomien nur einseitig erfolgt. Dadurch besteht die Gefahr einer insuffizienten Durchblutung mit nachfolgender Atrophie bis hin zur Nekrose. Zusätzlich ist die suffiziente Durchblutung der Mukosa und Submukosa essentiell für die Kontinenz [5].

Um die Versorgung durch eine zu enge Manschette nicht noch weiter zu kompromittieren, wurde zu einer größeren Manschette tendiert, was durch die Literatur als der gängige Weg bestätigt wird [6].

Durch diese intraoperativen Maßnahmen kam es zu keiner der gefürchteten Komplikationen und durch die Implantation des AUS zeigte sich eine verbesserte Kontinenz. Eine zufrieden stellende Sozialkontinenz konnte jedoch noch nicht erreicht werden.

Daher erfolgte, wie oben beschrieben, der Wechsel auf eine kleinere Manschettengröße. Die Methode des Wechsels lediglich einer Komponente des Systems ist bereits etabliert [7]. Die Notwendigkeit des kompletten Wechsels bestand nicht.

Auch die sofortige Aktivierung des Systems nach Manschettenwechsel, wie in unserem Fall ebenfalls erfolgt, geht nicht mit einer erhöhten Rate an Harnröhrenarrosionen einher [8].

Es muss erwähnt werden, dass wir bis dato kein suffizientes Follow-up unseres Patienten vorliegen haben, um die möglichen Langzeitkomplikationen, die bei jeder Sphinkterimplantationen auftreten können, zu evaluieren. Das Risiko dieser möglichen Komplikationen ist in unserem Fall theoretisch erhöht. Diese Ergebnisse bleiben abzuwarten, um eine Aussage über den Langzeitverlauf tätigen zu können.

Zusammenfassend ist das funktionelle Ergebnis sehr zufriedenstellend und dieser Fall ist einzigartig. Er zeigt, dass selbst mit initial äußerst verheerend komplikativen Verläufen die Implantation eines Sphinktersystems möglich ist und für den Patienten ein zufriedenstellendes Ergebnis ermöglicht.

## Fazit für die Praxis

- Ein künstlicher Schließmuskel ist und bleibt der Standard in der Versorgung der moderaten bis schweren Belastungsinkontinenz, vor allem nach radikaler Prostatektomie.
- Das Komplikationsmanagement bei implantiertem System ist herausfordernd und verlangt Erfahrung.
- Die perineale Urethrostomie stellt keine Kontraindikation zur Implantation einer AMS-800©-Sphinkterprothese dar, jedoch sind die individuellen anatomischen und funktionellen Besonderheiten der Patienten zu berücksichtigen:
  - Auf die korrekte Positionierung der Manschette muss geachtet werden (membranös), damit ein Sitzen ohne Missempfindungen am Damm ermöglicht wird und das System durch den perinealen Druck nicht beschädigt wird.
  - Die Manschettengröße darf durch die unidirektionale Blutversorgung der Harnröhre nicht zu klein gewählt werden.
- Unter Berücksichtigung der oben genannten Maßnahmen kann in ausgewählten Fällen, selbst bei verheerenden Verläufen in der Anamnese, die Sphinkterversorgung erfolgreich sein und die letzte gute Alternative zur Harnableitung darstellen.
